# Vital Relations

**Published:** 1874-01

**Authors:** P. H. Hayes


### VITAL RELATIONS--COLD AND HEAT.

P. H. HAYES, M. D.

There was once a time when the tropics extended to the poles, when there were both the plants and the animals of the torrid zone where now ice and snow forever cover the landscape. But this was from bottom heat, the earth was then cooling off into space, vastly more rapidly than now, and furnished this heat from her own bosom.

Now, the sun, thousands of times larger than the earth and two thousand times as hot as molten iron, is the source of all our heat. Yet so vast is its distance from our earth that we get only the one hundred millionth part of this heat, and what becomes of the rest we do not know. The earth absorbs heat very rapidly from the sun, but parts with it by radiation equally rapidly. This radiation is the source of the warmth of our own atmosphere, for it does not appear that the sun's rays warm the atmosphere by passing through it, and so it is found that as we ascend high mountains or rise in a balloon, or in other words, as we leave the surface of the earth, the atmosphere becomes rapidly colder, until, as philosophers say, the temperature of space is more than two hundred degrees below *zero.*—Into space, this void of inconceivable cold, the heat of the earth is radiated and lost. This radiation takes place in a clear night the faster. Fogs and clouds are like a mantle or blanket, holding the warmth and preventing frosts. In winter we are actually nearer the sun, but his oblique rays leave us colder than in summer. The heat and light of the sun go together, so we can never have a vigorous vegetation in our latitude in winter. And from the same causes, as we approach the poles at any season, the variety and vigor of all plant life constantly diminishes.

All objects upon the surface of the earth, like the earth itself, part with their heat, giving it off into that cold of *space* which no thermometer can measure. Yet only rocks and soils do this entirely passively. Plants produce, and being bad conductors and radiators, are able to retain a certain amount of heat.—Seeds produce heat while germinating, so when stored in mass as grain, and in the malting process, this heat becomes very apparent, sometimes rising as high as fifty degrees above the surrounding atmosphere. So also in the season of flowering, there is a marked increase of heat.

From the lowest animals upward there is a slowly increasing power to maintain an independent temperature, while in man himself this faculty is perfect. The marmot of the Alps keeps his temperature all right in summer, but when winter comes he rolls himself up in his nest very like a cocoon, and there he sleeps away the long night of winter, slowly burning up the fat of his body which he had stored up in the season of nuts and just before the winter set in. If the animal goes in poor, or the winter be very much prolonged, our poor marmot will die. In both the case of the plant and animal, oxygen disappears, uniting with carbon to produce the heat and forming carbonic acid. The power of producing heat in fishes is extremely low. Trout will freeze and then can be thus transported and recover life again.

The whale is properly a warm blooded animal and like ourselves maintains an even temperature of about 98°. The thick fat or blubber everywhere under his skin, first serves as a blanket and then as fuel to keep him warm. Were he not obliged to come to the surface so often to breathe, or as the sailors say to blow, it would be impossible to capture him. Ordinary fishes find for their low temperature all the air in the water which they need. Thus the gold fish we keep for pets in our houses seem all the time to be swallowing, but really they are just passing the water over their gills, the delicate fringes of which are so many lungs for the breathing of the air which the water contains, sufficient in amount for their very low degree of warmth. So low is the warmth of the water newt, that when kept for experiment it was not found necessary to feed the animal oftener than once in two months.

The temperature of our own bodies is 98° of Fahrenheit. More accurately it should be said that the internal parts of the body are 101°, while the arms and limbs are only 95°, and the feet still colder. In fevers this temperature often rises 7° to 10°, while in *Asthma* and the second stage of *Pneumonia*, the temperature will often run down as far below the natural standard.

In fevers there is a rapid oxygenation or burning up of the fatty parts of the body, and more rapid breathing to introduce this oxygen, while in *Asthma* and *Pneumonia* the heat runs down because the lungs are obstructed and the breathing space greatly diminished.

Nothing more markedly proves man's superior organization and life than his power to adapt himself to great extremes of temperature. Thus, in the tropics, the maximum degree of heat is 140°, while in the arctic regions the maximum cold is 50° to 60° below *zero*, a range of 200°. Even this does not express the whole truth, for though we do not know of any race or company of men who have long borne a climate colder than 50° or 60° below zero, yet on the other side, men have gone into ovens heated up to 200° and even 300° and remained while their food could be cooked there for them; nay, more, Chabert, the Fire King, when in this country entered into ovens heated up to 600°, and staid there a half hour. Of course the air in the oven must be absolutely dry. Had there been a pint of water spilled in this oven it would have killed the Fire King as promptly as though he had been shot. Thus man's power to bear extremes of heat and cold are greatly affected by the moisture of the air. The resident in Minnesota suffers less from the cold in his own State, where the mercury often falls until it is frozen, than he does in Philadelphia or even in Jacksonville, and the reason is because he is surrounded by an atmosphere which is perfectly *dry*, the moisture is all frozen out of it. And this dry air is the most perfect non-conductor of heat that can be found. On the other hand, your Philadelphian is enveloped in a heat-conducting and heat-absorbing *damp* air. The frequent south winds and mild days of the more southern climate relax the body and open the pores, and then when the cold change comes the body is without defence, and quickly chilled. The Minnesotian has a long, steadily cold winter, and the body is never unbraced or the skin relaxed by too warm weather. It must be remembered that motion in the air greatly diminishes the power of the body to resist cold. Thus, Dr. Kane said he could stand 70° below zero, if no wind were blowing, but if the wind was blowing it would make at least 30° difference in his power to endure cold. Clothing is to hold a wrapping of warm air next to the skin, but a wind drives this away. It must not be forgotten that there is another reason why damp air is always depressing, and that is because that a lungful of such an atmosphere contains just so much less real air as it contains moisture. Or in other words, the more moisture we breathe in the air the less air, and this is another cause of chilliness. Many men and women create for themselves artificially a bad climate and suffer all its sad consequences. I have seen a thousand times, men in their counting-rooms and offices working near their stoves and where the temperature was so high as certainly to relax the skin and open all its pores, and perhaps produce a slight perspiration. Let a person in this state go out into the cold, or even into a door-way, and they instantly get a check to perspiration, the body is chilled and coughs, colds, rheumatism, neuralgia and catarrh are the consequences. The same thing happens when, as is almost always done, both ladies and gentlemen sit in a warm church with all their wrappings on which they wore in from the cold. When they go out from the church they are surprised that their clothing does not prevent them from being chilly. The reason is plain, instead of a layer of *dry*, warm air next the skin, when they leave the church, it is a layer of warm vapor with which also their clothing has been dampened during their sitting. By this process the skin becomes relaxed, pale and highly sensitive. I have seen many such persons who have told me that cold was pain to them, that it made them cross and irritable, to say nothing of the diseases it induced.

It should be understood that all artificial warmth, beyond what is absolutely necessary for comfort, is weakening. Thus the habit of sitting with the feet upon the stove, of using hot bottles and bricks in bed and of excessive bed covering, weakens the power of the body to produce its own warmth, and makes the subject perpetually uncomfortable from chilliness in cold weather.

